# Activity-to-sedentary ratio provides novel insight into mortality reduction among male survivors of cardiovascular disease in the United States: national health and nutrition examination survey, 2007–2014

**DOI:** 10.1186/s12889-023-14978-4

**Published:** 2023-01-06

**Authors:** Yuanyuan Ding, Jiahao Chen, Mengying Niu, Qi Xiao, Hongqin Zhao, Xudong Pan, Xiaoyan Zhu

**Affiliations:** 1 Department of Neurology, The Affiliated Hospital of Qingdao University, Qingdao, 266000 Shandong China; 2 State Key Laboratory of Pathogen and Biosecurity, Beijing Institute of Microbiology and Epidemiology, Beijing, China; 3 Department of Epidemiology and Health Statistics, Public Health College, Qingdao University, Qingdao, China; 4 Department of Critical Care Medicine, The Affiliated Hospital of Qingdao University, Qingdao, 266000 Shandong China

**Keywords:** Activity-to-sedentary ratio, Cardiovascular disease, Mortality, Males, National Health and Nutrition Examination Survey

## Abstract

**Background:**

Lower physical activity and sedentary behavior have been identified as modifiable risk factors for cardiovascular disease (CVD). However, the quantitative, dose-response association between activity-to-sedentary ratio (ASR) and mortality is unknown.

**Methods:**

Prospective cohort studies with participants 50 to 80 years that reported the association between recreational physical activity, sedentary behavior, and all-cause mortality were included from the 2007 to 2014 United States National Health and Nutrition Examination Survey (NHANES) and followed through December 31, 2015. Cox or Weibull regression models and restricted cubic splines were used to determine the association between ASR and all-cause mortality.

**Results:**

Sixty deaths occurred among 498 CVD survivors, with a median of 56 months of follow-up. After accounting for all covariates, CVD survivors with an ASR between 0.21 and 0.57 (hazard ratio [HR], 0.47; 95% confidence interval [CI], 0.25–0.87) and those with an ASR more than 0.57 (HR, 0.40; 95% CI, 0.20–0.81) were at significantly lower risk for mortality than participants with an ASR <  0.21. Moreover, a nonlinear negative association and an L-shaped association were observed for the level of ASR with risk of mortality among CVD survivors (*P* for nonlinearity = 0.004). What’s more, adjusting for covariates, a statistically significant interaction (*P* for interaction = 0.016) between sex and ASR, an increase of ASR more than and equal to 0.18 was associated with a lower risk of mortality among males (HR, 0.23; 95% CI, 0.12–0.46).

**Conclusions:**

An negative correlation between ASR and mortality in CVD survivors, especially in males when ASR is more than 0.18. Our novel findings provide further insights into easing the global burden of deaths.

**Supplementary Information:**

The online version contains supplementary material available at 10.1186/s12889-023-14978-4.

## Introduction

Modifiable lifestyle factors, such as physical activity and sedentary behaviors, are strongly associated with the development and outcome of age-related cardiovascular disease (CVD), which is the leading cause of global mortality and disability [1–6]. According to the World Health Organization 2020 guidelines, physical activity and reducing sedentary behavior tend to outweigh the potential harms for all populations [7]. However, the total sitting time has increased among adults older than 65 in the United States from 2007 to 2014, according to the latest National Health and Nutrition Examination Survey (NHANES) data [8]. CVD survivors are likelier to engage in less physical activity [9].

Evidence for the consensus that physical activity is a robust preventive measure against CVD and all-cause mortality comes from accumulated studies [10–14]. The NHANES data showed that moderate-to-vigorous exercises such as walking, bicycling, and running reduced all-cause and CVD mortality [15]. Additionally, among 2370 individuals with CVD, increased total physical activity, leisure-time physical activity (LPA), and home-related or work-related physical activity were associated with lower mortality during 7 years of follow-up [16].

However, most previous studies have treated physical activity and sedentary behavior as separate entities that affect mortality [17, 18]. Recently, studies have focused on whether physical activity reduces or eliminates the harmful effects of being sedentary. In a meta-analysis using sixteen studies with more than one million participants, among inactive individuals, those who sat more than 8 h per day were at higher risk for all-cause mortality than those who sat less than 4 h per day (hazard ratio [HR], 1.27; 95% confidence interval [CI], 1.22–1.32). Active individuals sitting for more extended periods did not increase their mortality (HR, 1.04; 95% CI, 0.98–1.10); however, active individuals who watched television for more than 5 h increased their mortality (HR, 1.15; 95% CI, 1.05–1.27) [19]. It appeared that moderate-intensity physical activity, about four metabolic equivalents (METs), reduced the risk of death associated with prolonged sitting [19]. What’s more, a harmonized meta-analysis of nine prospective cohort studies from four countries analyzed the joint associations of accelerometer-measured physical activity and sedentary time (ST) with all-cause mortality; As a result, there was a considerable increase in the risk of death with lower levels of physical activity and more significant amounts of ST. [20]

While there is still a paucity of quantitative indicators to guide mortality prevention when considering the adverse effects of sedentary behavior based on previous knowledge, the activity-to-sedentary ratio (ASR) might be related to CVD events and has the potential to be a quantitative indicator of cardiovascular events and mortality prevention. Therefore, this study aimed to investigate a quantitative, dose-response association between ASR and mortality in CVD survivors.

## Methods

### Study population

NHANES, a program of the National Center for Health Statistics, is a prominent, nationally representative, cross-sectional survey conducted throughout the United States over 2 years. Details of the survey design and collected information are described on the website [21]. Public data from the NHANES 2007–2008, NHANES 2009–2010, NHANES 2011–2012, and NHANES 2013–2014 were selected for this study. We included CVD survivors (defined with a history of congestive heart failure, coronary heart disease, angina pectoris, heart attack, and stroke) aged 50 to 80 years with PAQ data and no cancer. National center for health statistics (NCHS) Research Ethics Review Board approval was waived for this analysis because of the publicly available and de-identified data.

### Assessment of LPA, ST, and ASR

This survey uses the Physical Activity Questionnaire (PAQ), which is based on the Global Physical Activity Questionnaire and includes questions related to daily activities, leisure activities, and sedentary activities.

ST was assessed by asking participants how much time (minutes) they usually spend sitting or reclining on a typical day (not including sleeping but including time spent sitting at a desk, sitting with friends, reading, playing cards, watching television, using a computer and traveling in a car, bus, or train).

LPA was assessed by asking participants if they participate in and how many days, and how much time per day they perform any recreational vigorous-intensity sports or moderate-intensity sports, fitness, or recreational activities that cause significant increases in the breathing rate or heart rates such as running or basketball for at least ten minutes continuously.

A MET is the amount of oxygen required to sustain resting metabolism [22]. The NHANES suggested that the MET score of vigorous LPA was 8.0 and that of moderate LPA was 4.0. The following formula was used to calculate daily energy expenditure: (frequency) × (average duration of each session) × (MET score) [23].

We used the NHANES self-reported LPA and ST to measure the ASR, a new variable defined as the amount of LPA (MET-hours/day) divided by the total ST (hours) that reflects the interaction between LPA and ST.

### Assessment of potential confounders

Based on previous literature, potential confounders included demographic data (age, sex, race/ethnicity, education, and income), physical examinations results (body mass index [BMI], blood pressure), questionnaires results (medical conditions, PAQ, smoking, diabetes), and laboratory test results [19]. Hispanic/Mexican, non-Hispanic white, non-Hispanic black, and others were the categories of race/ethnicity. Education was divided into three categories (less than high school, high school or equivalent, and college or more). The family poverty-to-income ratio was categorized as 0.00–0.99 (below the poverty level) and ≥ 1.00 (equal to or above the poverty level) [21]. Three categories of smoking status were identified (never, former, and current). Participants with a history of hypertension, systolic blood pressure ≥ 130 mmHg, diastolic blood pressure ≥ 80 mmHg, or using antihypertension medications were classified as having hypertension [24, 25]. We identified diabetes as a history of diabetes or glycated hemoglobin ≥6.5% or fasting blood glucose ≥7.0 mmol/L or two-hour postprandial blood glucose level ≥ 11.0 mmol/L according to the Oral Glucose Tolerance Test (OGTT) or the use of hypoglycemic drugs or insulin [26].

### Mortality assessment

The primary outcome was all-cause mortality. Participants were followed-up according to the National Death Index interview until December 31, 2015 [27].

### Statistical analysis

According to the NCHS, to combine 8 years of data from 2007 to 2014, we create an eight-year weight variable by dividing the 2-year weights (WTMEC2YR) by the number of two-year cycles in the world analysis, the formula is as follows: if sddsrvyr in (5,6,7,8) then MEC8YR = 1/4 * WTMEC2YR [28].

For continuous variables, normality tests were performed. We calculated the weighted mean ± standard error (SE) for normally distributed data. We used the weighted median and interquartile range (IQR) for skewed distribution data. Categorical variables were summarized as weighted percentages±SE. Differences between groups were compared using Mann-Whitney’s U test adjusted for sampling weights for continuous variables and the chi-square test (design-based F-statistic) for categorical variables [29, 30].

Because there were no definite thresholds for ASR, we divided the surviving participants into tertiles.

We included an interaction term for treatment with the logarithm of time in the Cox models to test the proportionality assumption. According to our preliminary analysis, there was a violation of the proportionality assumption. However, the Weibull distribution was appropriate based on graphical plots assessing Weibull assumptions (p ≠ 1) (Fig. S1A). Similar to the Cox model, the Weibull model assumes the baseline hazard as the Weibull distribution instead of being nonparametric. The parametric survival curves were estimated using Weibull survival models and the log-rank test was used to test the equality of the survivor function across groups. Results of the Weibull regression are displayed as HR at 95% CIs. An ASR <  0.21 was used as the reference. Model 1 was adjusted for age and sex only. Model 2 was adjusted for age, sex, race/ethnicity, education, poverty-to-income ratio, smoking status, and BMI. In model 3, we further adjusted for hypertension and diabetes based on model 2.

Additionally, we estimated the dose-response relationships of ASR with the outcomes and predicted the HR for mortality for each respondent using Cox regression; we adjusted for the same variables mentioned previously and fit restricted cubic splines and 95% CIs for four points (the 5th, 35th, 65th, and 95th percentiles) by treating the amount of ASR as a continuous variable [31]. The 5th percentile was used as a reference, and a test for departure from linearity was performed using the testparm command for all spline components except the first spline component to fit the restricted cubic spline [32].

To test the robustness and potential variations in different sex subgroups, we repeated survival models stratified by sex (male and female), and *P*-values of the multiplicative interaction terms were reported as measures of interaction. ​In the male and female groups, parametric hazard models with Weibull distributions were used (p ≠ 1) (Fig. S1B, C). Given the lack of sample size for subgroup analysis, we used ROC analysis to find a cut-off or median value to convert the continuous variable ASR to categorical variables. Sensitivity analysis was performed by repeating the parametric hazard model with the Weibull distribution using data excluding participants who died in the first year of follow-up to reduce the possibility of spurious associations (Fig. S1D, E, F).

In this study, we followed the guidelines for strengthening the reporting of epidemiological observational studies [33]. No missing values were included in the final analysis. STATA 16.0 (Stata Corp, College Station, TX) was used for statistical analyses. All statistical tests were two-sided, and *P* < 0.05 was considered statistically significant.

## Results

### Participant characteristics

**Fig. 1:**
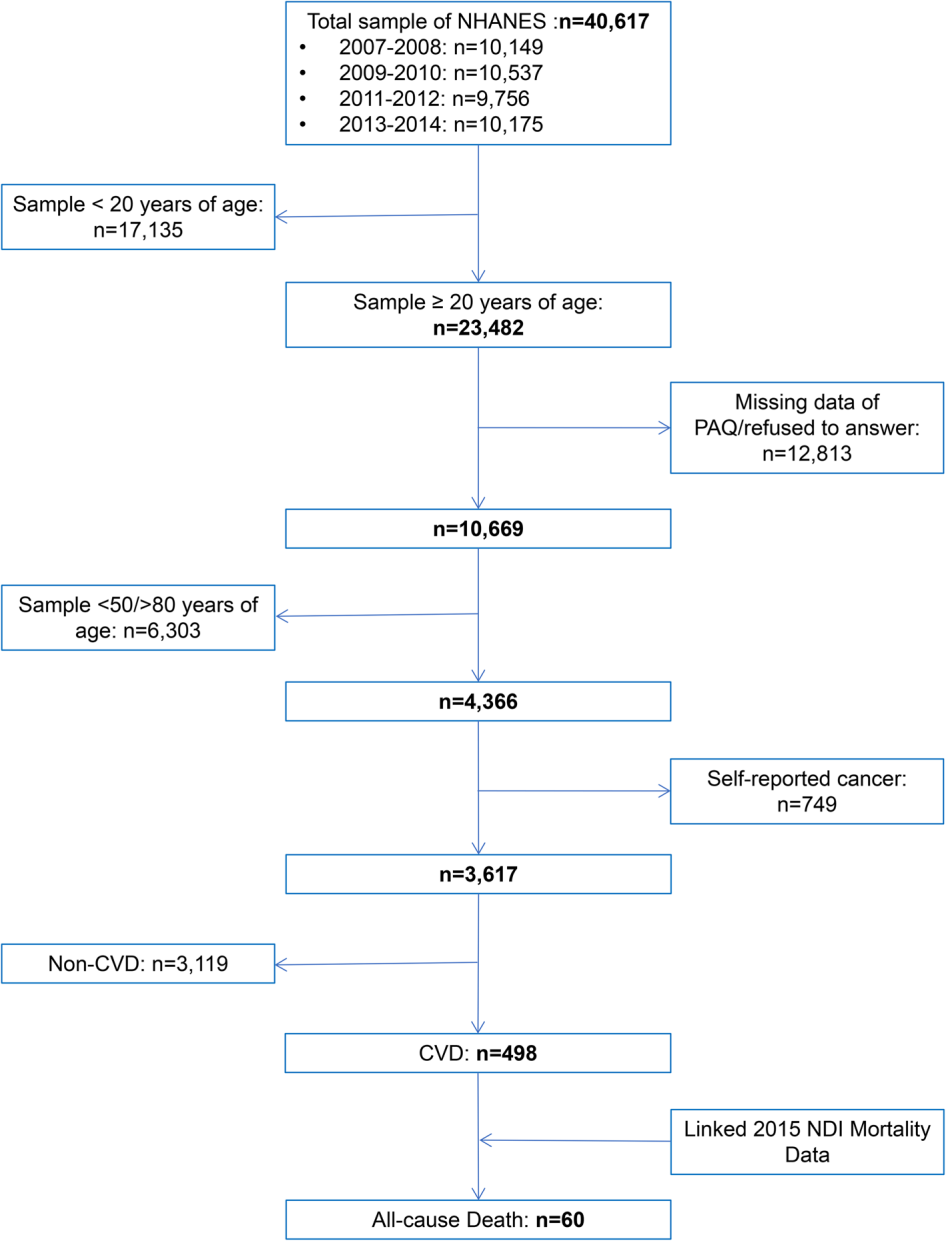
Flowchart of sample selection among NHANES study participants

Of 498 CVD survivors between 50 and 80 years of age at the time of the survey with PAQ data, during a weighted mean of 56 months of follow-up, a total of 60 deaths, or 12% of deaths were recorded (Fig. 1). In Table 1, those CVD survivors were grouped by mortality status. In participants who did not survive to follow-up, the median ASR level was 0.20, which was relatively low compared to those who were still alive, who had an ASR of 0.36. Those who died were more likely to be older, more sedentary, and less physically active. However, no statistical difference is observed between them.

**Table 1:** Baseline characteristics^a^ of adults aged 50–80 years by mortality status from NHANES 2007–2014

Characteristic	Total(***n*** = 498)	Deceased(***n*** = 60)	Survivor(***n*** = 438)	***P*** value
Age, years, mean ± SE	66.9 ± 0.1	74.0 ± 0.5	66.2 ± 0.1	0.005
Sex, % ± SE				0.361
Male	63.8 ± 0.4	70.6 ± 5.9	63.1 ± 0.2	
Female	36.2 ± 0.4	29.4 ± 5.9	36.9 ± 0.2	
Race/ethnicity, % ± SE				0.560
Hispanic/Mexican	6.4 ± 0.3	8.7 ± 4.5	6.2 ± 0.7	
Non-Hispanic white	80.0 ± 0.7	73.2 ± 2.4	80.7 ± 1.0	
Non-Hispanic black	8.0 ± 1.1	9.6 ± 0.8	7.9 ± 1.2	
Other	5.5 ± 0.2	8.5 ± 5.5	5.2 ± 0.4	
Education, % ± SE				0.502
Less than high school	16.3 ± 0.2	18.3 ± 6.6	16.1 ± 0.5	
High school or equivalent	25.7 ± 2.8	32.4 ± 6.3	25.0 ± 2.8	
College or above	58.0 ± 3.0	49.3 ± 10.5	58.9 ± 2.3	
PIR, % ± SE				0.990
0.00–0.99	10.7 ± 2.6	10.6 ± 3.6	10.7 ± 3.2	
1.00 or above	89.3 ± 2.6	89.4 ± 3.6	89.3 ± 3.2	
Smoking status, % ± SE				0.413
Never	44.8 ± 2.5	32.5 ± 2.9	46.0 ± 2.7	
Former	43.6 ± 0.5	55.6 ± 7.3	42.4 ± 0.9	
Current	11.6 ± 2.7	11.8 ± 5.0	11.6 ± 3.5	
BMI (Kg/m^2^), mean ± SE	29.2 ± 0.2	29.4 ± 0.3	29.2 ± 0.2	0.693
Hypertension, % ± SE				0.528
Yes	77.0 ± 2.4	82.1 ± 8.2	76.4 ± 1.9	
No	23.0 ± 2.4	17.9 ± 8.2	23.6 ± 1.9	
Diabetes, % ± SE				0.050
Yes	27.2 ± 1.2	39.3 ± 3.3	26.0 ± 1.3	
No	72.8 ± 1.2	60.7 ± 3.3	74.0 ± 1.3	
ST (h/d), mean ± SE	5.9 ± 0.1	6.5 ± 0.3	5.8 ± 0.1	0.169
LPA (MET-h/day), Median (IQR)	1.7(1.86)	1.1(1.43)	2.0(2.29)	0.579
ASR, Median (IQR)	0.36(0.54)	0.20(0.65)	0.36(0.61)	0.259

*Abbreviation*s: *BMI* Body mass index, *PIR* Poverty-to-income ratio, *ST* Sedentary times, *PA* Physical activities, *ASR* Activity sedentary ratio, *SE* Standard error, *IQR* Interquartile range

^a^Data are shown as the weighted mean or frequency (SE) or Median (IQR) as appropriate

*P*-values indicate statistically significant differences if *P* < 0.05

### ASR and all-cause mortality

**Fig. 2:**
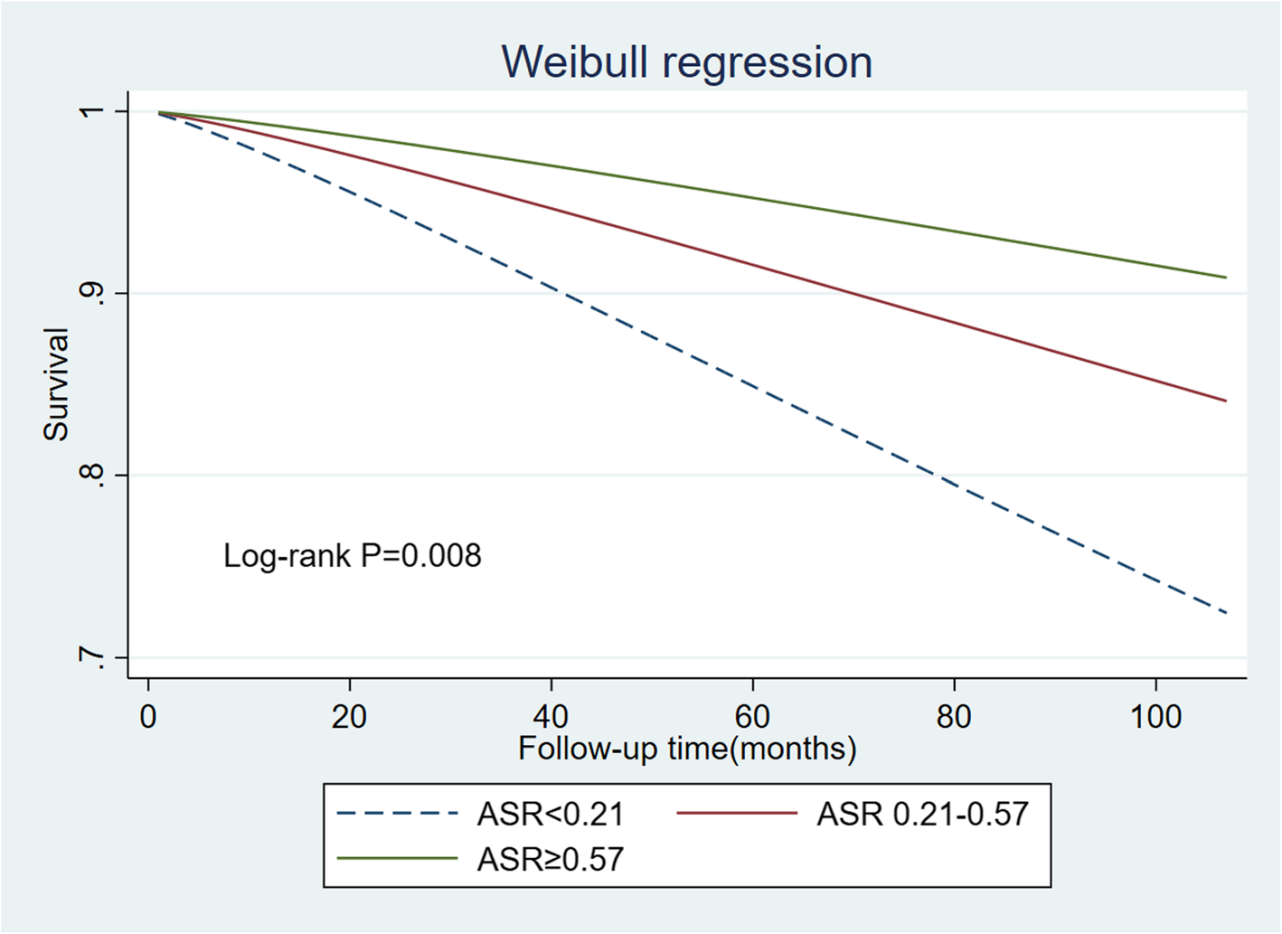
Survival functions from Weibull parametric model fitting for different ASR groups

Figure 2 shows the survival curves based on the assumption of a Weibull distribution. From the perspective of different ASR levels, the risk of death is relatively lower for elevated ASR levels. Table 2 presents the HRs for ASR from the Weibull proportional hazard model. Adjusted HRs for the Weibull model were 0.47(95% CI 0.25–0.87) and 0.40(95% CI 0.20–0.81). This analysis suggests that higher ASR may protect against all-cause deaths.

**Table 2:** Weighted Hazard ratio (95% CI) of All-cause Mortality across tertiles of ASR. Multivariable-Adjusted analysis *n* = 498

	Deceased events/participants (%)	Hazard ratio (95% CI)			
		Unadjusted	Model 1^**a**^	Model 2^**b**^	Model 3^**c**^
Tertile1(< 0.21)	30/175 (17.1)	1[Reference]	1[Reference]	1[Reference]	1[Reference]
Tertile2(0.21–0.57)	16/157(10.2)	0.54(0.29–0.98) ^*^	0.51(0.28–0.94) ^*^	0.48(0.26–0.90) ^*^	0.47(0.25–0.87) ^*^
Tertile3(≥ 0.57)	14/166 (8.4)	0.41(0.21–0.79)^**^	0.45(0.23–0.87) ^*^	0.42(0.21–0.84) ^*^	0.40(0.20–0.81) ^*^

^a^Model 1: adjusted + age + sex

^b^Model 2: model 1 + race/ethnicity + education + PIR + smoking status+ BMI

^c^Model 3: model 2 + hypertension + diabetes

^*^P < 0.05, ^**^P < 0.01

**Fig. 3:**
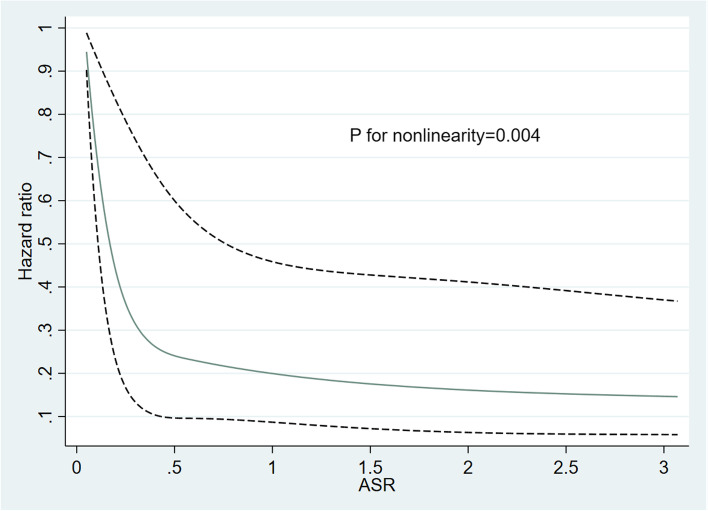
Dose-response association of ASR and all-cause mortality in CVD survivors. Restricted cubical splines for the adjusted hazard ratio. Knots were set at the 5th, 35th, 65th and 95th percentile. The 5th percentile was used as a reference. The solid blue line is the multivariable adjusted hazard ratio and the black dashed line shows the 95% CI. The model was adjusted for age, sex, race/ethnicity, education, PIR, smoking status, BMI, hypertension and diabetes

The estimated dose-response association between ASR and all-cause mortality was evaluated using a nonlinear spline model. CVD survivors had an L-shaped association between ASR and mortality risk. The risk of all-cause mortality decreases sharply until the ASR reaches approximately 0.57; after that, the risk tended to decrease slowly (*P* for nonlinearity = 0.004) (Fig. 3).

The ASR cut-off value from ROC analysis in males was 0.18 in Fig. S2. In Table 3, adjusting for covariates, a statistically significant interaction (*P* for interaction = 0.016) between sex and ASR, an increase of ASR more than and equal to 0.18 was associated with a lower risk of mortality among males (HR, 0.23; 95% CI, 0.12–0.46).

**Table 3:** The weighted Hazard ratio (95% CI) of All-cause Mortality uses the cut-off value or Median of ASR in CVD survivors according to sex

	Deceased events/participants (%)	Unadjusted HR (95% CI)	Adjusted HR (95% CI)
Male			
ASR ≥ 0.18	22/243(9.0)	0.29 (0.13–0.65) ^**^	0.23(0.12–0.46) ^***^
Female			
ASR ≥ 0.29	7/75(9.3)	0.71(0.21–2.36)	1.42(0.30–6.63)
*P* for interaction		0.029	0.016

The model was adjusted for age or sex and race/ethnicity + education + PIR + smoking status+ BMI + hypertension + diabetes

^**^P < 0.01, ^***^P < 0.001

### Sensitivity analysis

Removing deaths occurring in the first year of follow-up, repeating analyses showed that during a weighted mean of 56 months of follow-up, a total of 51 deaths among 489 participants, or 10.4% of deaths were recorded. An ASR of 0.21–0.57 and an ASR ≥ 0.57 were significantly negatively correlated with all-cause mortality and when the ASR increased, the HR decreased substantially regardless of whether an adjustment for confounding was performed (Table S1). Among males, ASRs higher than 0.18 were associated with a lower risk of mortality (Table S2).

## Discussion

Our study is the first to explore a quantitative, dose-response association between ASR and mortality among 498 CVD survivors in the United States. ASR was used as a new variate to analyze the association with all-cause mortality by combining the amounts of LPA and ST. Our findings showed a negative correlation between ASR and mortality in CVD survivors, especially in males when ASR is more than 0.18. ​Therefore, for male survivors of CVD, ASR may serve as an essential quantitative indicator of mortality prevention.

The results of our study support previous studies, to some extent, suggesting that reducing sedentary time along with increasing physical activity may provide mortality benefits [34, 35]. In general terms, if males with CVD were sedentary for 10 hours, according to the minimum cut-off value for analysis, approximately at least 1.8 MET-hours of LPA would be necessary to alter the detrimental effect of leisure-time sedentary behavior, equivalent to approximately at least 27 minutes of moderate LPA or 13.5 minutes of vigorous LPA might reduce 77% risk of all-cause mortality. The results corroborate recommendations from the United States Physical Activity Guidelines that short sitting durations and high levels of physical activity are positively associated with mortality risk [36]. However, there are some differences between our study and previous studies. First, the significance of our threshold or this quantitative ratio measure is observed only among male CVD survivors. To some extent, this is consistent with previous research suggesting that people with CVD may benefit more from physical activity [37]. The sex differences in our study could be attributed to sex differences in CVD and mortality and lifestyle, such as cigarette smoking being more common in males than females [38].

The potential biological mechanisms underlying the association between ASR and the all-cause mortality risk of those with CVD are largely unknown and may be partially explained by atherosclerosis. Atherosclerosis, the primary cause of CVD, involves a combination of inflammation and immunity [39]. On the one hand, physical activity is considered an effective strategy for avoiding atherosclerosis. Animal experiments have shown that physical activity reduces the incidence of atherosclerosis in mice by modulating C5 levels and innate immunity to reduce chemotaxis [40]. Furthermore, physical activity mainly targets epicardial adipose tissue, which can reduce the progression of atherosclerosis and endothelial damage by reducing the release of lipids in epicardial tissue [41]. Physical activity may also play an essential role in stress reduction in managing cardiovascular disease [42]. Conversely, sedentary behavior may modify the vascular structure and function, resulting in stiffness, increased intima-media thickness, and decreased endothelial function [43]. Sedentary behavior may also increase the risk of CVD associated with increased BMI, cardiorespiratory fitness, blood pressure, insulin resistance, and blood lipids [43]. Mechanistic studies should be performed to clarify the roles of physical activity and ST in the long-term health of individuals with CVD.

There are some potential limitations of this study. Firstly, it is possible that residual confounding factors, measured or unmeasured, may exist even after accounting for various potential confounders. As HDL (cholesterol) and triglycerides and alcohol consumption, were not taken into account because the sample size was insufficient due to excessive missing values that were not at random, and this needs to be investigated in future studies. When BMI is used as an indicator of body adiposity, it is related to age and gender, there may be selection bias of confounding factors [44]. Secondly, questionnaires may be biased by recall errors and response bias [45]. There was heterogeneity in the results between studies that used self-reported physical activity and those that used objectively measured physical activity, such as accelerometers [45]. However, the results from PAQ may be advantageous due to the limited overall agreement, measurement method, and study dimension [46]. Besides, self-reported sedentary behaviors do not always reflect actual sitting time. Due to the present study’s focus on LPA and sedentary behavior, work-related physical activity and sitting time analyses are lacking to reflect the standard population. Further analysis considering the ratio of work-related physical activity to sedentary behavior could add further insight. On the other hand, considering the influence of seasonal effects on physical activity, a single time point assessment (2007–2014 baseline) may preclude the examination of longitudinal effects in this study, and our conclusions are based on observational studies, which may be subject to some unavoidable biases. Study participants with partial cycles had a relatively short follow-up. This may lead to a slight underestimation of the potential impact of adherence to healthy lifestyle behaviors on mortality. In addition, there are some unavoidable exclusion biases due to missing information, and the effect of disease perception on the outcome due to hospitalization rates, which deserve our attention in future studies.

## Conclusion

In short, our study suggests that a higher ASR is associated with lower mortality risk among male CVD survivors. To reduce the global burden of death, avoiding prolonged sedentary hours and increasing the ratio of LPA to sedentary hours could be significant strategies.

## Supplementary Information

**Additional file 1: Figure S1.** The distribution plot of ln(-ln(S(t))) relative to ln(t) for assessing Weibull assumptions. **Figure S2.** The cut-off value was determined for ASR using the standard ROC curve in male group. **Table S1.** Weighted Hazard ratio (95% CI) of All-cause Mortality across tertiles of ASR, excluding participants who died in the first year of the follow-up. Multivariable-Adjusted analysis *n*=489. **Table S2.** The weighted Hazard ratio (95% CI) of All-cause Mortality uses the cut-off value or Median of ASR in CVD survivors according to sex, excluding participants who died in the first year of the follow-up. Multivariable-Adjusted analysis *n*=489.

## Data Availability

The datasets generated and analyzed during the current study are available on the NHANES website: https://wwwn.cdc.gov/nchs/nhanes/Default.aspx

## Abbreviations

LPA: Leisure-time physical activity
ASR: Activity-to-sedentary ratio
CVD: Cardiovascular disease
ST: Sedentary time
HR: Hazard ratio
CI: Confidence interval
NHANES: National Health and Nutrition Examination Survey
PAQ: Physical Activity questionnaire
MET: Metabolic equivalent
BMI: Body mass index
IQR: Interquartile range
SE: Standard error
