# Supplementary material for: Activity-to-sedentary ratio provides novel insight into mortality reduction among male survivors of cardiovascular disease in the United States: national health and nutrition examination survey, 2007–2014
Source: BMC Public Health. 2023 Jan 6;23:35. doi: 10.1186/s12889-023-14978-4 (PMC9817385; doi:10.1186/s12889-023-14978-4)
Supplement: Supplementary file 1 — Additional file 1: Figure S1. The distribution plot of ln(-ln(S(t))) relative to ln(t) for assessing Weibull assumptions. Figure S2. The cut-off value was determined for ASR using the standard ROC curve in male group. Table S1. Weighted Hazard ratio (95% CI) of All-cause Mortality across tertiles of ASR, excluding participants who died in the first year of the follow-up. Multivariable-Adjusted analysis n=489. Table S2. The weighted Hazard ratio (95% CI) of All-cause Mortality uses the cut-off value or Median of ASR in CVD survivors according to sex, excluding participants who died in the first year of the follow-up. Multivariable-Adjusted analysis n=489. [file 12889_2023_14978_MOESM1_ESM.docx]

**Additional file 1 — supplement tables and figures**

- 1. **Supplementary Figures**

**Figure S1. The distribution plot of ln(-ln(S(t))) relative to ln(t) for assessing Weibull assumptions**

**Figure S2. The cut-off value was determined for ASR using the standard ROC curve in male group.**

**
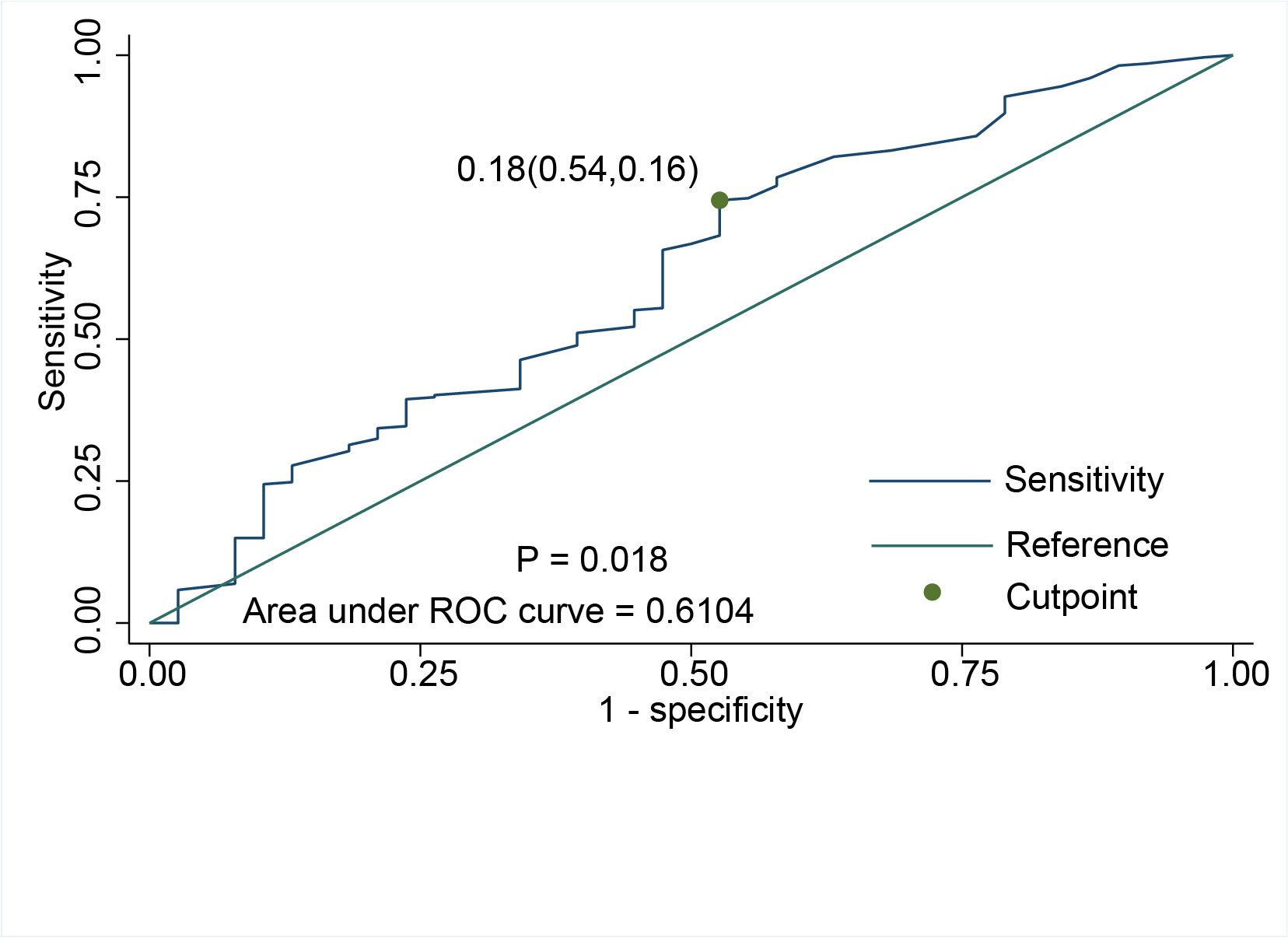
**

**1.2 Supplementary Tables**

**Table S1. Weighted Hazard ratio (95% CI) of All-cause Mortality across tertiles of ASR**, **excluding participants who died in the first year of the follow-up. Multivariable-Adjusted analysis n=489.**

|  |  | **Hazard ratio (95% CI)** | | | |
| --- | --- | --- | --- | --- | --- |
|  | **Deceased events/participants (%)** | **Unadjusted** | **Model 1^a^** | **Model 2^b^** | **Model 3^c^** |
| ASR |  |  |  |  |  |
| < 0.21 | 28/173 (16.1) | 1[Reference] | 1[Reference] | 1[Reference] | 1[Reference] |
| 0.21-0.57 | 12/153 (7.8) | 0.40(0.17-0.91) ^*^ | 0.41(0.18-0.90) ^*^ | 0.40(0.18-0.88) ^*^ | 0.37(0.17-0.78) ^**^ |
| ≥ 0.57 | 11/163(6.7) | 0.24(0.10-0.56) ^**^ | 0.24(0.10-0.56) ^**^ | 0.25(0.11-0.56) ^**^ | 0.22(0.10-0.50) ^***^ |

Abbreviation: NA, not applicable

^a^ Model 1: adjusted + age + sex.

^b^ Model 2: model 1 + race/ethnicity + education + PIR + smoking status+ BMI.

^c^ Model 3: model 2 + hypertension + diabetes.

^*^*P* < 0.05, ^**^*P* < 0.01, ^***^*P* < 0.001.

**Table S2. The weighted Hazard ratio (95% CI) of All-cause Mortality uses the cut-off value or Median of ASR in CVD survivors according to sex**, **excluding participants who died in the first year of the follow-up. Multivariable-Adjusted analysis n=489.**

**.**

|  | **Deceased** **events/participants (%)** | | **Unadjusted HR (95% CI)** | | **Adjusted HR (95% CI)** | |
| --- | --- | --- | --- | --- | --- | --- |
| Male | |  | |  | | |
| ASR ≥ 0.18 | 18/239(7.5) | | 0.24 (0.10-0.55) ^**^ | | 0.18(0.09-0.38) ^***^ | |
| Female | |  | |  | | |
| ASR ≥ 0.29 | 4/72(5.5) | | 0.33(0.08-1.31) | | 0.33(0.05-2.33) | |
| *P* for interaction |  | | 0.019 | | | 0.009 |

Models was adjusted for age or sex and race/ethnicity + education + PIR + smoking status+ BMI + hypertension + diabetes.

^**^*P* < 0.01, ^***^*P* < 0.001
